# Preparation of Novel Nano-Sized Hydrogel Microcapsules via Layer-By-Layer Assembly as Delivery Vehicles for Drugs onto Hygiene Paper

**DOI:** 10.3390/polym10030335

**Published:** 2018-03-19

**Authors:** Junrong Li, Jing Zou, Huining Xiao, Beihai He, Xiaobang Hou, Liying Qian

**Affiliations:** 1 State Key Laboratory of Pulp and Paper Engineering, South China University of Technology, Guangzhou 510640, China; lljrr@scut.edu.cn (J.L.); spew1992@163.com (J.Z.); ppebhhe@scut.edu.cn (B.H.); 2 Department of Chemical Engineering, University of New Brunswick, Fredericton, NB E3B 5A3, Canada; xhou1@unb.ca; 3 Department of Environmental Science and Engineering, North China Electric Power University, Baoding 071003, China

**Keywords:** hydrogel microcapsules, layer-by-layer assembly, sodium alginate, polyethyleneimine, ciprofloxacin

## Abstract

Hydrogel microcapsules are improved transplantation delivery vehicles for pharmaceuticals by effectively segregating the active ingredients from the surroundings and delivering them to a certain target site. Layer-by-layer (LbL) assembly is an attractive process to fabricate the nano-sized hydrogel microcapsules. In this study, nano-sized hydrogel microcapsules were prepared through LbL assembly using calcium carbonate nanoparticles (CaCO_3_ NPs) as the sacrificial inorganic template, sodium alginate (SA) and polyethyleneimine (PEI) as the shell materials. Ciprofloxacin was used to study the encapsulation and release properties of the hydrogel microcapsules. The hydrogel microcapsules were further adsorbed onto the paper to render antimicrobial properties. The results showed that the mean size of the CaCO_3_ template was reduced after dispersing into sodium *n*-dodecyl sulfate (SDS) solution under sonication. Transmission electron microscope (TEM) and atomic force microscope (AFM) revealed that some hydrogel microcapsules had a diameter under 200 nm, typical creases and collapses were found on the surface. The nano-sized PEI/SA hydrogel microcapsules showed high loading capacity of ciprofloxacin and a sustained release. PEI/SA hydrogel microcapsules rendered good antimicrobial properties onto the paper by the adsorption of hydrogel microcapsules, however, the mechanical properties of the hygiene paper were decreased.

## 1. Introduction

Encapsulation is a promising route for the development of improved transplantation delivery vehicles for pharmaceuticals by effectively segregating the active ingredients from the surroundings for the given period of time and delivering them to a certain target site from harsh environment factors, such as pH, salts, and shear stress. To ensure adequate exchange of gases, nutrients, and metabolic products between encapsulated pharmaceuticals and the external environment, hydrogel is a kind of ideal shell material for its ability to uptake a large amount of water or biological fluids and tunable microcapsule diffusivity. Some stimuli-responsive hydrogel shell can control the drug release, even provide “on-off” pulsatile drug release with a sharp, rapid sensitive response [1]. Therefore, hydrogel microcapsules are attracting lots of interest in their applications such as drug loading and targeting release, cell cultivation and transportation, biocatalyst [2], etc. Several methods were applied to fabricate the hydrogel microcapsules such as microfluidics [3,4,5,6,7,8], Layer-by-layer (LbL) assembly [9,10,11,12,13], in situ electrospinning [14,15], electrospraying [16,17,18], or electro-jetting [19]. Clearly each technique has its own strengths and its suitable applications. Microfluidics has emerged as a powerful tool for generating hydrogel microcapsules without damages to encapsulated cells, however, the obtained hydrogel microcapsules are usually dispersed in an oil phase and difficult to be extracted for further biomedical applications [20]. Moreover, it is feasible for some approaches (microfluidics or 3D printing-assisted microfluidics [6]) to realize continuous production of functional hydrogel microcapsules with a size of several hundred micro meters [21], the preparation of nano-sized hydrogel microcapsules is still challenging. LbL polyelectrolyte assembly is an attractive process for coating templates and subsequently removing the core to fabricate hydrogel microcapsules [22,23,24,25]. LbL polymeric microcapsules possess many significant advantages in their mechanical performance and the possibility to control shell composition, thickness, diameter, density, and permeability [26]. A variety of particulate substrates including inorganics [27] and polymeric materials [28] have been used as templates for hydrogel microcapsules formation.

The driving force of LbL self-assembly is the complementary interactions, e.g., electrostatic attraction, hydrogen bonding or covalent bonding, on the substrates. The types of layering materials and the number of layers are the main factors that determine the structure and properties of hydrogel microcapsules, such as the stability and the release rate. The strong ionic interaction between cationic and anionic polyelectrolytes in LbL deposition made the structure of the hydrogel microcapsules stable and has the ability to regulate the release rate. Polysaccharides are usually used to prepare the shell materials of hydrogel microcapsules due to their high biocompatibility, biodegradability, and low cost. Alginate is widely used in preparation of hydrogel microcapsules because of its rapid ionic gelation with divalent cations [8,29]. However, hydrogel microcapsules of alginate alone have a relatively slow degradation profile and it does not promote efficient cell attachment [30]. In an attempt to overcome these restrictions of alginate, some polymers were combined with it to fabricate the complex hydrogel microcapsules [31,32,33,34]. Chitosan is one of the most commonly used components [16,17,32,33], however, the alginate/chitosan hydrogel microcapsules have some limitations that need to be improved, such as mechanical strength of the shell membrane, influence of deacetylation degree of chitosan [35], and fibrous overgrowth on the surface of the implanted hydrogel microcapsules [36,37]. Polyethyleneimine (PEI), a branched cationic polymer with the high charge density, is a good candidate to form LbL film with anionic polymers. It is also used to fabricate the hydrogel microcapsules by LbL assembly with anionic polyelectrolytes for its proper ionic strengths and charge densities [38]. PEI is considered in this study to be combined with alginate to fabricate hydrogel microcapsules to load the medicine.

Herein, we demonstrated the preparation of nano-sized hydrogel microcapsules through LbL assembly, using calcium carbonate nanoparticles (CaCO_3_ NPs) as the template, sodium alginate (SA) and PEI as the wall material. In order to obtain steady nano-sized template suspension, an anionic surfactant was added and the influence of the concentrations on the particle size was investigated. The ionic interaction between amino groups of PEI and the carboxyl residues of alginate led to a stable PEI/SA polyelectrolyte complex membrane by layer by layer assembly. Ciprofloxacin, as an antibacterial medicine, was used as the model drug to study the encapsulation and release properties of the hydrogel microcapsules. The hydrogel microcapsules were further adsorbed onto paper to render antimicrobial properties and the properties of the hygiene paper were also investigated.

## 2. Materials and Methods

### 2.1. Chemicals

Sodium alginate (SA) (15–25 cP), polyethyleneimine (PEI) (*M_w_* = 2.5 × 10^4^), hydrochloric acid (HCl), phosphate buffer saline (PBS) and ciprofloxacin were all purchased from Sigma-Aldrich (Oakville, ON, Canada). Ethylenediaminetetraacetic acid disodium salt (EDTA) and sodium *n*-dodecyl sulfate (SDS) were purchased from Alfa Aesar (Tewksbury, MA, USA). Calcium carbonate nanoparticles (CaCO_3_ NPs) (15–40 nm) were purchased from SkySpring Nanomaterials, Inc. (Houston, TX, USA). *E. coli* (ATCC11229) and agar were purchased from Huankai Microbial Sci. & Tech. Co. Ltd. (Guangzhou, China). All of the chemicals were of laboratory reagent grade and used without further purification.

### 2.2. Preparation of PEI/SA Microcapsules

**Scheme 1:**
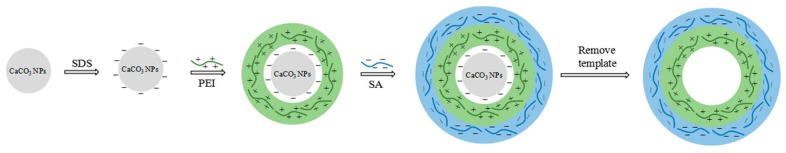
Fabrication of nano-sized and polyethyleneimine (PEI)/sodium alginate (SA) hydrogel microcapsules.

Nano-sized PEI/SA microcapsules were prepared through LbL assembly of SA and PEI using CaCO_3_ NPs as sacrificial templates. The fabrication procedure was shown in Scheme 1. Firstly, CaCO_3_ NPs were immersed in 50 mL of SDS solution and dispersed under sonication for 30 min, then 50 mL of PEI aqueous solution (1 mg/mL) was added to the template suspension and stirred for 30 min to allow first PEI layer to be absorbed onto the surface of CaCO_3_ NPs. Excess SDS and PEI were removed by centrifugation (4800 rpm, 15 min) and the templates were washed with deionized water for three times. Then negatively charged SA and positively charged PEI solution were assembled alternatively on the CaCO_3_ NPs by dispersing the templates into 100 mL of polyelectrolyte solutions (1 mg/mL), the templates were incubated for 20 min, centrifuged and washed to obtain the desired bilayers of polyelectrolytes. In order to remove the template, CaCO_3_ NPs loaded with polyelectrolytes were dispersed into 0.1 M EDTA solution and incubated for 24 h. The PEI/SA hydrogel microcapsules were obtained after three centrifugation/washing cycles and drying at room temperature, the hydrogel microcapsules used in this paper were fixed as 2 bilayers of the polyelectrolytes.

### 2.3. Characterization of the Hydrogel Microcapsules

Mean particle size and zeta potential were determined with Brookhaven’s zeta potential instruments (Brookhaven Instruments Corporation, New York, NY, USA). Atomic force microscope (AFM) was used to examine the morphologies of the hydrogel microcapsules. AFM images were obtained with a Nanoscope IIIa (Veeco Instruments, Camarillo, CA, USA) in tapping mode using a silicon probe (NP-S20, Veeco Instruments, Camarillo, CA, USA) with settings of 512 pixels/line and 1 Hz scan rate. Transmission electron microscope (TEM) and energy dispersive spectrometer (EDS) were used to analyze the element composition of samples. The hydrogel microcapsules were observed with a JEOL 2010 scanning TEM instrument (JEOL Ltd., Akishima, Tokyo, Japan) operated at an accelerating voltage of 200 keV. A drop of 20 μL ca. 0.01 wt. % hydrogel microcapsules dispersion was transferred to a carbon-coated copper grid using a pipette. The grid was air-dried at room temperature overnight.

### 2.4. Loading and Release of Ciprofloxacin

The drug solution was prepared by dissolving various amounts of ciprofloxacin into 0.1 M HCl. In order to study the effect of drug concentrations on the loading capacity, 10 mg of hydrogel microcapsules were incubated in 50 mL of ciprofloxacin solution for 6 h. As for the effect of loading time on loading capacity, the PEI/SA hydrogel microcapsules (10 mg) were incubated in ciprofloxacin solution of 0.5 mg/mL. After being incubated, the ciprofloxacin-loaded hydrogel microcapsules were centrifuged (4800 rpm, 15 min), washed for 2 times with deionized water and dried. The loading amount of ciprofloxacin was calculated from the difference of ciprofloxacin concentrations in the supernatant which were determined by measuring the remained ciprofloxacin in the supernatant at 316 nm by UV spectra (Genesys 10 s, Thermo Electron Corporation, Waltham, MA, USA). The drug content is the percentage of the loaded ciprofloxacin to the PEI/SA hydrogel microcapsules. For the releasing experiment, 5 mg of ciprofloxacin-loaded PEI/SA hydrogel microcapsules were dispersed into 100 mL 0.1 M HCl solution and the cumulative release ratio of the ciprofloxacin from hydrogel microcapsules was also monitored at 316 nm of UV absorbance. All the results were averaged from 3 parallel experiments. The drug loading content and cumulative release ratio was calculated according to formula (1) and (2):

Drug loading content = *m*_1_/*m*_2_ (1)

Cumulative release ratio = *m_t_*/*m*_1_ (2)

where *m*_1_ is the weight of loaded ciprofloxacin, *m*_2_ is the weight of microcapsules, *m_t_* is the weight of released ciprofloxacin.

### 2.5. Preparation and Characterization of Hydrogel Microcapsules Absorbed Paper

A circular filter paper sample (1 g) was incubated in hydrogel microcapsules suspensions with a certain amount of cationic PEI for 24 h with shaking, then vacuum dried for further characterization. Tensile and tearing strength of paper samples were tested by tensile strength tester (CE062, L&W, Kista, Stockholm, Sweden) and tearing strength tester (009, L&W, Kista, Stockholm, Sweden) respectively. Thermal behaviors of paper samples were measured using thermogravity analysis tester (Q-500, TA Instruments Inc., New Castle, DE, USA). Temperature raised from 25 °C to 600 °C under N_2_ flow, heating rate 10 °C/min.

Antimicrobial properties of paper samples were tested by shaking flask method [39]. 1 g of paper scraps were sterilized and mixed with 5 mL *E.* *coli* (2.5 × 10^7^ CFU mL^−1^) at 200 rpm and 37 °C for 4 h. After shaking, 1 mL of solution was diluted with PBS solution and cultured on agar medium at 37 °C for 24 h, then the number of bacteria colonies was counted and the antimicrobial efficiency was calculated.

## 3. Results

### 3.1. Dispersion of CaCO_3_ Nanoparticles

Normally, CaCO_3_ microparticles were used as templates to assemble the hydrogel microcapsules [40] and CaCO_3_ NPs were seldom to be used because they were ready to agglomerate in aqueous solution [41,42,43] due to the small size and high surface energy. In order to reduce agglomeration and obtain uniform sized CaCO_3_ templates, SDS was used as a surfactant to control the size of the templates with sonication before the deposition of multi polymer layers.

**Figure 1:**
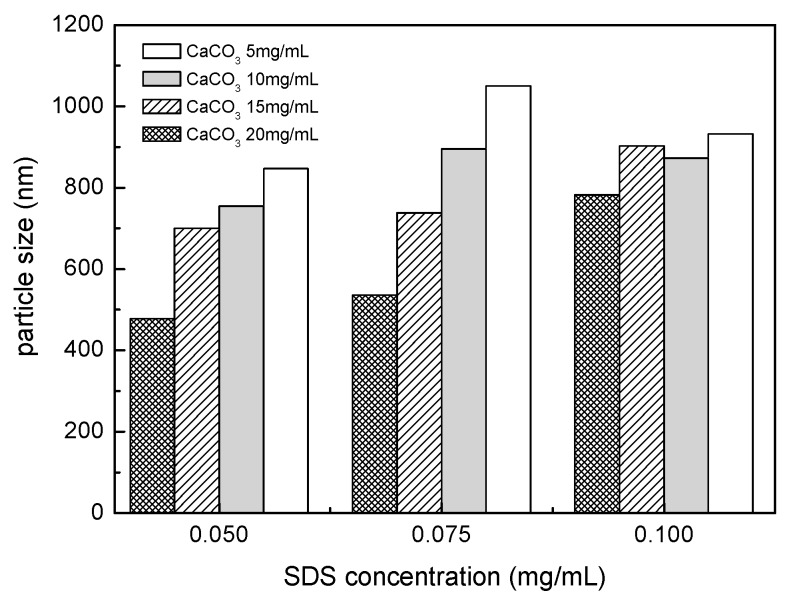
Influence of sodium *n*-dodecyl sulfate (SDS) and CaCO_3_ concentrations on the particle size of templates.

Effects of concentrations of SDS and CaCO_3_ on the mean particle size were shown in Figure 1. It could be seen that with the increase of SDS concentration, the size of CaCO_3_ NPs increased and became similar for various CaCO_3_ concentrations because the excess SDS can envelope the small particles into bigger ones. The concentration of CaCO_3_ in solution showed less influence on template size at higher SDS concentration. The dispersion of CaCO_3_ NPs in solution might be attributed to the interaction between SDS and the particle surface [44]. The dispersion conditions were set at an SDS concentration of 0.05 mg/mL, CaCO_3_ concentration of 20 mg/mL to get a CaCO_3_ template particle size of 478 nm which were applied in the fabrication of hydrogel microcapsules for loading of ciprofloxacin.

### 3.2. Fabrication of PEI/SA Hydrogel Microcapsules

After uniformly dispersing CaCO_3_ into SDS solution, nano-sized PEI/SA hydrogel microcapsules were fabricated using CaCO_3_ NPs as sacrificial templates. The electrostatic force between negatively charged SA and positively charged PEI was the main force during the fabrication of multi polymer layers [45]. Zeta potential of the nano-sized hydrogel microcapsules showed charge reversal in each layering step during the fabrication. The original CaCO_3_ templates had a negative zeta potential of −9.62 mV due to the existence of SDS and hydroxyl group on the surface [46], and the surface potential of the hydrogel microcapsules was observed to change regularly from +25 mV for PEI to −17 mV for SA during their assembling on the surface of the templates, indicating the formation of the desired LbL wall architecture. The polyelectrolyte multilayer wall might serve multiple purposes, including providing a protective shell for encapsulated biomolecules and making the structures useful for biological and biomedical applications such as sustained release [47,48].

**Figure 2:**
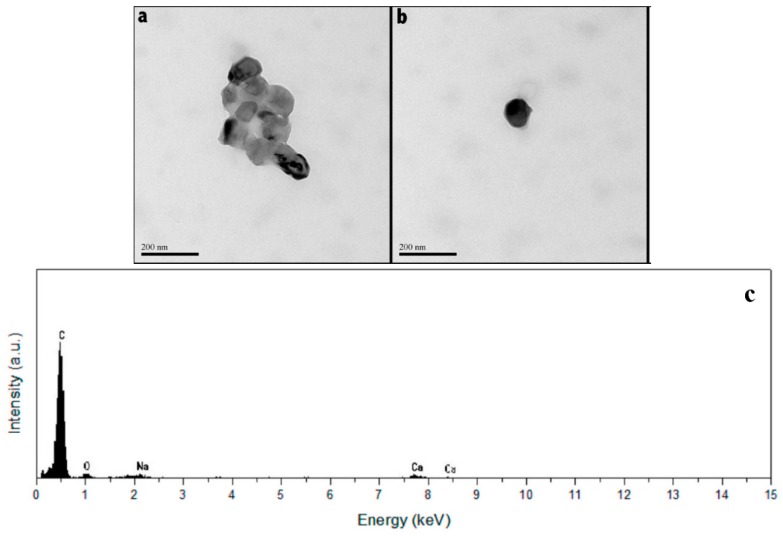
Transmission electron microscope (TEM) of nano-sized PEI/SA hydrogel microcapsules (**a**) agglomerated hydrogel microcapsules; (**b**) single microcapsule; and (**c**) energy dispersive spectrometer (EDS) image of PEI/SA hydrogel microcapsules.

TEM and EDS were used to investigate the morphology and composition of the nano-sized PEI/SA hydrogel microcapsules after the removal of the CaCO_3_ templates. The mean size of primary CaCO_3_ NPs used in this work was between 15 nm and 40 nm and they were ready to agglomerate to larger particles in aqueous solution. Therefore, the concentrations of SDS and CaCO_3_ NPs were manipulated to obtain the relatively stable CaCO_3_ templates with the mean size of 478 nm. The process of the removal of the CaCO_3_ templates made the hydrogel microcapsules collapse to some extent which reduced the size of some microcapsules compared to the templates. More importantly, the aggregated CaCO_3_ templates were very loose structures and the LbL film tended to absorb on the surface of individual particles when the aggregated templates were incubated in the polyelectrolyte solutions. The electrostatic repulsion of charges in the LbL film facilitated the disaggregation of the templates into smaller ones with a diameter under 200 nm observed in Figure 2b. The darker areas in the hydrogel microcapsules represented higher elements content, revealing the typical morphology on the surface.

Figure 2c showed the element composition of PEI/SA hydrogel microcapsules. The strongest peak represented carbon from the polymer layers, oxygen and sodium were from sodium alginate layers, and a spot of calcium was from residual templates.

**Figure 3:**
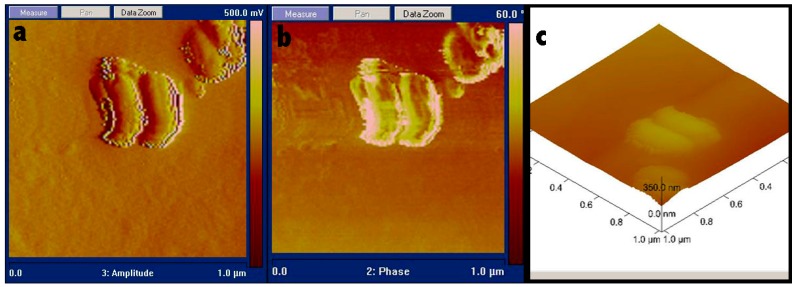
Morphology of PEI/SA hydrogel microcapsules revealed by atomic force microscope (AFM). (**a**) amplitude image; (**b**) phase image; (**c**) 3-D image.

AFM images exhibited similar morphology of the PEI/SA hydrogel microcapsules to TEM. As shown in Figure 3, agglomeration of nano-sized hydrogel microcapsules was found (Figure 3a), the fold and crease on the surface of the hydrogel microcapsules were clearly revealed (Figure 3b). Some hydrogel microcapsules presented a flat shape with the height of 50 nm while the diameter was 200 nm after removal of the templates because of the collapse in the center (Figure 3c).

### 3.3. Loading and Releasing of Ciprofloxacin

**Figure 4:**
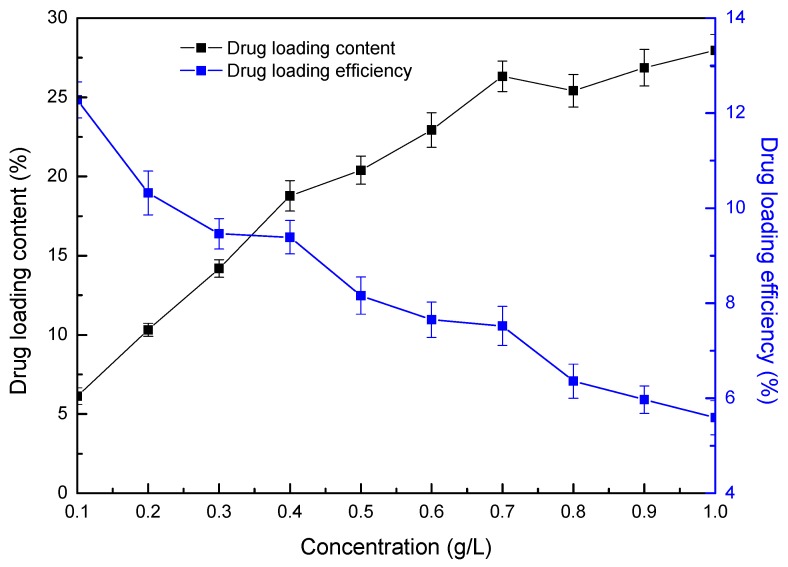
Effect of drug concentration on the loading content and loading efficiency.

The effects of drug concentration on the drug loading content and load ratio were shown in Figure 4. In general, the drug loading content of PEI/SA hydrogel microcapsules increased from 6.14% to 28.0% when ciprofloxacin concentration increased from 0.1 to 1.0 g/L and this increase slowed down at a higher concentration of ciprofloxacin, however, the drug loading efficiency decreased with the increasing drug concentration. The high loading and nonlinear increase of drug content could be attributed to the spontaneous deposition of ciprofloxacin into the PEI/SA hydrogel microcapsules. Some positively charged substances had been reported to spontaneously accumulate inside the hydrogel microcapsules with the driving force of electrostatic attraction between the incorporated positively charged substances and negatively charged complex formed during the preparation of the hydrogel microcapsules [49]. Since ciprofloxacin was positively charged [50] and PEI/SA microcapsules carried negative charge, the spontaneous deposition of the drug onto the surface and into the inner cell of the hydrogel microcapsules could be greatly increased.

**Figure 5:**
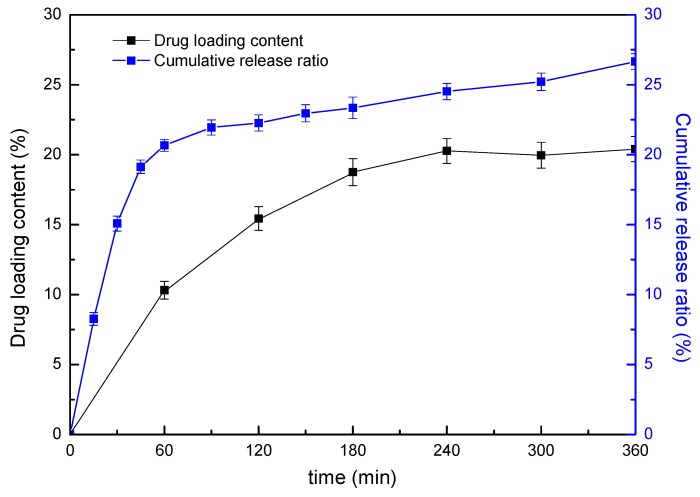
Effect of time on the drug loading content and cumulative release ratio.

As shown in Figure 5, the drug loading content increased rapidly in the first 4 h and reached the maximal content of 20.3%, and then it reached equilibrium. In the first few hours, electrostatic interaction between the negative SA layer and the positive ciprofloxacin facilitated the adsorption of the drug onto the surface of the hydrogel microcapsules. Meanwhile, spontaneous deposition of some substances into the polyelectrolyte multilayer capsules was a general phenomenon for polyelectrolyte capsules [51] and it provided a facile pathway to load various water-soluble substances, especially positively charged species into the polyelectrolyte capsules [52]. The molecular size of ciprofloxacin was smaller than 1–2 nm and the polyelectrolytes layer-by-layer film was a kind of porous structure, therefore, the drug could permeate into the inner cavity of the hydrogel microcapsules easily due to the concentration differences of ciprofloxacin between the outside and inside of the hydrogel microcapsules [52]. With more drugs entered into the hydrogel microcapsules, the driving force to loading became smaller and drug content reached the equilibrium which was the total amount of adsorbing on the surface and entrapping in the cell of hydrogel microcapsules.

The cumulative release curve of ciprofloxacin from PEI/SA hydrogel microcapsules was also shown in Figure 5. The initial burst release was observed within the first 1 h and the release ratio reached 20.7% of total loaded drug which caused by quick release of both the adsorbed drug from the surface and the encapsulated drug from the inner cavity because of the largest imbalance between the capsule interior and the bulk [52]. PEI/SA hydrogel microcapsules showed sustained release behavior with a cumulative release amount of 26.7% at 6 h.

### 3.4. Incorporation of Hydrogel Microcapsules onto the Paper

Incorporation of drug loaded hydrogel microcapsules onto paper is an efficient approach to render antimicrobial property to the paper product. Table 1 showed the influence of hydrogel microcapsules concentration on the antimicrobial efficiency of paper. PEI is a polymer abundant in amino groups of primary, secondary, and tertiary types at the typical ratio of 1:2:1; and it shows limited antimicrobial activity for bearing some cationic charges but good selectivity toward bacteria [53]. Therefore, the drug unloaded hydrogel microcapsules control samples showed certain antimicrobial efficiency which increased from 22.1% to 40.6% with the increase of concentration of hydrogel microcapsules. Ciprofloxacin is a common antibiotic with excellent antimicrobial activity and the loading of ciprofloxacin into hydrogel microcapsules increased the antimicrobial efficiency of samples significantly. The antimicrobial efficiency of drug loaded samples increased from 46.7% to 86.3% and the antimicrobial efficiency of drug loaded samples was much higher than microcapsule samples under each concentration gradient, indicating that ciprofloxacin was successfully released from hydrogel microcapsules and paper.

**Figure 6:**
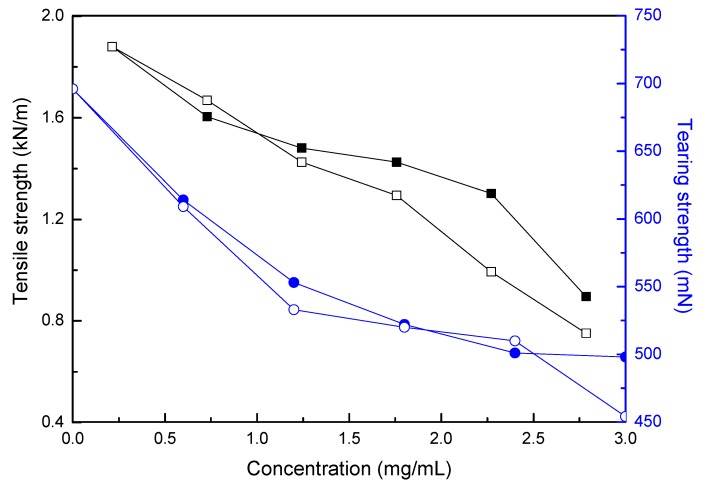
Influence of microcapsule concentration on the tensile and tearing strength of paper (blue: tensile strength; black: tearing strength; solid: drug loaded samples; hollow: drug unloaded samples).

Tensile and tearing strength represent the mechanical properties of paper. Figure 6 showed the influence of hydrogel microcapsules concentration on the tensile and tearing strength of paper after the absorption. Both tensile and tearing strength decreased with the increase in concentration. Tensile strength decreased from 1.88 kN/m to 0.90 kN/m and tearing strength decreased from 696 mN to 498 mN for drug loaded samples; tensile strength decreased from 1.88 kN/m to 0.75 kN/m and tearing strength decreased from 696 mN to 454 mN for drug unloaded samples. These decreases represented the weakening of binding force between fibers because that hydrogel microcapsules could block hydrogen bonding between fibers. In addition, in the process of preparation, the paper is wetted and then dried. In general, the process itself leads to a decline in the strength of the paper.

**Figure 7:**
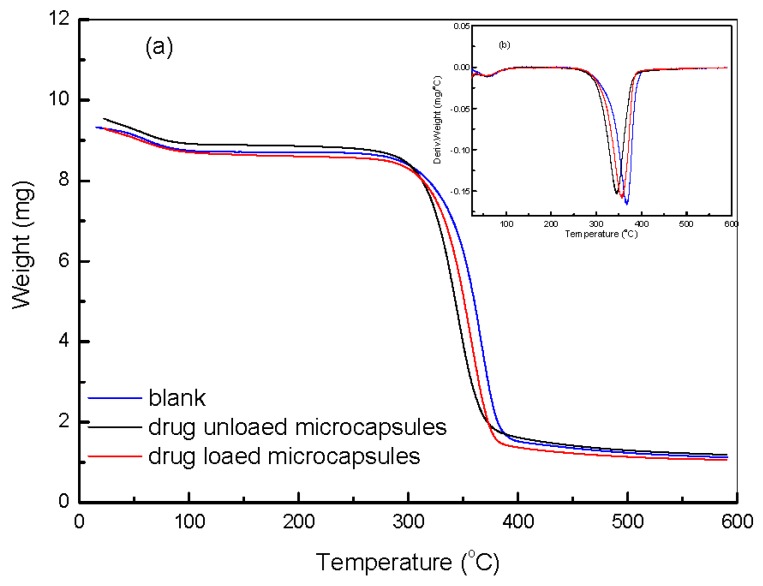
Thermogravity of hydrogel microcapsules loaded paper (**a**) and first derivative curve (**b**).

Figure 7 showed the thermogravity curves of blank paper samples, hydrogel microcapsules samples, and drug loaded hydrogel microcapsules samples. There were two stages of weight loss, first stage was from 25 °C to 110 °C for each sample, the loss ratio was about 6%, which was attributed to the loss of free and bound water. For blank paper samples, the second stage was from 270 °C to 410 °C with the loss ratio of 80%, for hydrogel microcapsules sample and drug loaded hydrogel microcapsules sample, the second stage was from 255 °C to 390 °C with the loss ratio of 83%, indicating that the absorption of hydrogel microcapsules decreased the thermal stability of paper because the hydrogen bonding between cellulose fibers was deteriorated. Figure 7b presented the first derivative curves of Figure 7a, the drug loaded hydrogel microcapsules sample showed higher maximum weight loss temperature (365 °C) than drug unloaded hydrogel microcapsule samples (350 °C), indicating that drug loaded hydrogel microcapsules possessed higher thermal stability. Ciprofloxacin was a positively charged molecule and a portion of the loaded ciprofloxacin adsorbed on the surface of the hydrogel microcapsules. It readily formed an electrostatic interaction between hydrogel microcapsules and negative-charged cellulose fibers, which compensated the decrease of thermal stability resulted by the deteriorated hydrogen bonding. Therefore, the drug loaded hydrogel microcapsules exhibited higher thermal stability, compared to drug unloaded one.

## 4. Conclusions

Nano-sized hydrogel microcapsules were prepared through layer-by-layer (LbL) assembly, using calcium carbonate nanoparticles (CaCO_3_ NPs) as the sacrificial inorganic template, polyethyleneimine (PEI) and sodium alginate (SA) as the wall materials. The results showed that SDS could act as a dispersant to greatly reduce the agglomeration of CaCO_3_ NPs in solution. Zeta potential analysis showed charge reversal in each layering step during the fabrication of PEI/SA hydrogel microcapsules. TEM and AFM showed that the prepared hydrogel microcapsules had a diameter under 200 nm after removing the templates. The nano-sized PEI/SA hydrogel microcapsules showed a high loading capacity of ciprofloxacin. Ciprofloxacin release from hydrogel microcapsules took on a sustained release and the PEI/SA hydrogel microcapsules adsorption paper showed good antimicrobial efficiency. The mechanical properties and the thermal stability of paper were decreased after the absorption of hydrogel microcapsules.

## Figures and Tables

**Table 1:** Influence of microcapsule concentration on the antimicrobial efficiency of paper.

Microcapsule Concentration (mg/mL)	Antimicrobial Efficiency (%)	
	Drug Unloaded Samples	Drug Loaded Samples
0.5	22.1 ± 1.1	46.7 ± 2.1
1.0	31.3 ± 1.5	58.4 ± 2.6
1.5	37.8 ± 1.7	69.8 ± 2.8
2.0	39.6 ± 1.9	73.4 ± 3.1
2.5	40.8 ± 1.6	82.1 ± 4.1
3.0	40.6 ± 1.5	86.3 ± 3.9
